# Potential Application of *Lonicera japonica* Extracts in Animal Production: From the Perspective of Intestinal Health

**DOI:** 10.3389/fmicb.2021.719877

**Published:** 2021-08-09

**Authors:** Xiaopeng Tang, Xuguang Liu, Jinfeng Zhong, Rejun Fang

**Affiliations:** ^1^State Engineering Technology Institute for Karst Desertfication Control, School of Karst Science, Guizhou Normal University, Guiyang, China; ^2^Hunan Polytechnic of Environment and Biology, College of Biotechnology, Hengyang, China; ^3^College of Animal Science and Technology, Hunan Agricultural University, Changsha, China

**Keywords:** *Lonicera japonica* extract, intestinal microorganisms, intestinal immunity, intestinal health, animal production

## Abstract

*Lonicera japonica* (*L. japonica*) extract is rich in active substances, such as phenolic acids, essential oils, flavones, saponins, and iridoids, which have a broad spectrum of antioxidant, anti-inflammatory, and anti-microbial effect. Previous studies have demonstrated that *L. japonica* has a good regulatory effect on animal intestinal health, which can be used as a potential antibiotic substitute product. However, previous studies about intestinal health regulation mainly focus on experimental animals or cells, like mice, rats, HMC-1 Cells, and RAW 264.7 cells. In this review, the intestinal health benefits including antioxidant, anti-inflammatory, and antimicrobial activity, and its potential application in animal production were summarized. Through this review, we can see that the effects and mechanism of *L. japonica* extract on intestinal health regulation of farm and aquatic animals are still rare and unclear. Further studies could focus on the regulatory mechanism of *L. japonica* extract on intestinal health especially the protective effects of *L. japonica* extract on oxidative injury, inflammation, and regulation of intestinal flora in farm animals and aquatic animals, thereby providing references for the rational utilization and application of *L. japonica* and its extracts in animal production.

## Introduction

The animal intestinal tract is the direct place for the communication between the internal environment and the external environment, and is an important defense line for animals to maintain the homeostasis of the internal environment (Tang et al., 2016). A healthy gut is essential for the growth and development of animals. However, the intestinal epithelium homeostasis of animals is affected by numerous factors, such as bacterial infection, endotoxin challenge, weaning stress, and oxidative stress, leading to intestinal damage and intestinal barrier function dysfunction (Campbell et al., 2013; Yin et al., 2014; Zhu H. et al., 2018). Traditionally, antibiotics are generally used as growth and health promoters, which have achieved certain achievements and promoted the development of animal husbandry (Barton, 2014). However, the abuse of antibiotics in livestock and poultry feeds will destroy the intestinal microecological balance, and lead to the resistance of bacteria (Hashemi and Davoodi, 2011; Looft et al., 2014), which would bring serious negative effects on human health and environmental safety. Therefore, the use of antibiotics as intestinal microecological regulator is no longer popular. Exploring new antibiotic substitutes to regulate intestinal microflora and to maintain the intestinal health of animals is an urgent task in the field of animal nutrition in the post-antibiotic era.

Plant extract is a complex mixture of compounds. It has been reported to possess multiple bioactivities such as antioxidant (Liu et al., 2018), anti-inflammatory (Wu et al., 2017), anti-microbial (Kavoosi et al., 2013), and immune regulation (Boskabady et al., 2013). Plant extracts have been used for centuries in traditional medicine and as food preservatives, and more recently have been studied as possible feed additives used in animal nutrition due to their multiple biological functions (Kim et al., 2012; Yejun et al., 2019). *Lonicera japonica* (*L. japonica*) extract is extracted from *L. japonica* Thunberg, a medicine food homologous herb rich in organic acids, volatile oils, flavonoids, iridoids, and saponins (Shang et al., 2011; Fan et al., 2019; Li R. et al., 2020), which have high value of health benefits. *Lonicera japonica* extract is widely used in pharmacological preparations, cosmetics, food, and animal husbandry because of its diverse pharmacological effects such as antioxidant, anti-microbial, antiviral, antitoxic, antiseptic, and anti-inflammatory properties (Kang et al., 2010; Park et al., 2012; Yejun et al., 2019). The application of *L. japonica* extract in animal production mainly focuses on pigs (Liu W. et al., 2016), beef cattle (Yejun et al., 2019), dairy cows (Ma et al., 2020b; Zhao et al., 2020), broiler (Müştak et al., 2015), laying hens (Long et al., 2018), *Penaeus monodon* (Chen et al., 2013), grass carp (Meng et al., 2019), and olive flounder (Dharaneedharan et al., 2016). From the results of these studies, *L. japonica* extract can function as a potential alternative antibiotic in animal feeds. However, the studies of the impacts of *L. japonica* extract on the intestinal health of animals are scattered in different pieces of literature, and little research could aggregate these findings into a single review. Therefore, the objective of this study was to review the effects of *L. japonica* extract on intestinal health and summarize its application in animal production.

## Bioactive Compounds of *L. Japonica* Extract

*Lonicera japonica*, also known as Japanese honeysuckle, Jin YinHua or Ren Dong, belongs to the member of the *Caprifoliaceae* family, is a perennial deciduous shrub native to East Asia and spread throughout Argentina, Brazil, Mexico, Australia, New Zealand, and American (Kim et al., 2015). Traditionally, the flower bud of *L. japonica*, which has been listed in the Chinese Pharmacopeia as *L. japonica* Flos, is a traditional Chinese medicine that reportedly has antioxidant, anti-inflammatory, antibacterial, antiviral, antitumor, and antidiabetic properties (Liu Z. et al., 2016; Shi et al., 2016; Wang et al., 2017), which has been widely used for preventing and treating influenza, cold, fever, and infections (Kashiwada et al., 2013; Ge et al., 2018; Fang et al., 2020). *Lonicera japonica extract* is extracted from *L. japonica*, has complicated chemical composition. So far, more than 300 chemical compounds have been isolated from and identified from *L. japonica*, and the major compositions are phenolic acids, essential oils, flavones, saponins, and iridoids (Shang et al., 2011; Li et al., 2017, 2019; Li R. et al., 2020).

### Phenolic Acids

There are more than 49 kinds of phenolic acids in *L. japonica*, which is mainly composed of chlorogenic acid (CGA) derivatives and cinnamic acid derivatives (Duan et al., 2018; Li et al., 2019; Li and Han, 2020; Qiu et al., 2021). A total of 27 CGA have been isolated and identified from *L. japonica*, such as CGA, neochlorogenic acid (NGC), isochlorogenic acid A, isochlorogenic acid B, isochlorogenic acid C, etc. (Iwahashi et al., 1986; Chang and Hsu, 1992; Peng et al., 2000; Lee et al., 2010; Seo et al., 2012; Yu et al., 2015; Duan et al., 2018; Li et al., 2019; Liu et al., 2020; Wang H. et al., 2020). About 16 cinnamic acid derivatives, like caffeic acid (CA), 1-*O*-caffeoylquinic acid, trans-cinnamic acid, trans-ferulic acid, caffeic acid methyl ester, and so on, have been isolated and identified from *L. japonica* (Iwahashi et al., 1986; Chang and Hsu, 1992; Choi et al., 2007; Jeong et al., 2015; Yu et al., 2015; Duan et al., 2018; Li et al., 2019). Other phenolic acids including 2,5-dihydroxybenzoic acid-5-O-β-D-glucopyranoside, vanillic acid, vanillic acid 4-O-β-D-(6-O-benzoyl glucopyranoside), vanillic acid-4-O-β-D-(6-O-benzoyl pyranoside), and protocatechuic acid (Choi et al., 2007; Lee et al., 2010; Li et al., 2019) were also identified from *L. japonica*. Among them, CGA and CA are the two most studied compounds in *L. japonica*, which have confirmed to possess potent activities of anti-inflammation, antioxidant, and antibacterial (Hsu et al., 2016; Hou et al., 2017; Li et al., 2019; Li R. et al., 2020). In particular, CGA is the most abundant phenolic acid in *L. japonica*, and it has been used as a marker to characterize the chemical qualities of *L. japonica* (Tzeng et al., 2014; Chen et al., 2017a; Li et al., 2019).

### Essential Oils

Essential oils are one of the bioactivity components of *L. japonica*, which mainly composed of acids, aldehydes, alcohols, ketones, and their esters (Li et al., 2019). They exist in the aerial parts of *L. japonica*, flower (fresh and dry), leaves, and vines with a different content and composition (Shang et al., 2011). Vukovic et al. (2012) showed that the main constituents in the flowers fraction were (Z,Z)-farnesole (16.2%) and linalool (11.0%), the main constituents in the leaves fraction were hexadecanoic acid (16.0%) and linalool (8.7%), and the main constituents in the stems were hexadecanoic acid (31.4%). Essential oils in *L. japonica* are also affected by different habitats. Du et al. (2015) identified 35 volatile constituents in *L. japonica* from Guangxi Zhuang Autonomous Region (China), mainly including methyl linolenate, n-hexadecanoic acid, and *ɛ*-muurolene, and 18 volatile constituents in LJF from Hunan province (China), mainly including n-hexadecanoic acid, linoleic acid, and *ɛ*-curcumene. Essential oils, the most kinds of bioactivity component in *L. japonica*, have important pharmacological effects, and have been used in cosmetics, spices, and other industries widely (Wang L. et al., 2016). It also suggests that the characterization of the volatile compounds could be used as an indicator of the identity and the quality of *L. japonica* (Cai et al., 2013).

### Flavonoids

Flavonoids are secondary metabolites and widely exist in natural plants including *L. japonica* (Han et al., 2016; Li R. et al., 2020; Liu et al., 2020), a group of natural or synthetic compounds containing parent cyclic structures and their O- and C-glycosylated derivatives with structural diversity (Rauter et al., 2018). Up to now, about 52 flavonoids have been isolated from *L. japonica*, which is mainly composed of flavonols (12 kinds) and flavones (36 kinds), and most of them are glycosides (Li et al., 2019). The flavonols mainly include rutin, quercetin, isoquercitrin, astragalin, Quercetin 3-O-hexoside, and so on (Chang and Hsu, 1992; Choi et al., 2007; Lee et al., 2010; Seo et al., 2012; Ge et al., 2019). The main flavones including cynaroside, luteolin, chrysoeriol 7-O-neohesperidoside, chrysoeriol 7-O-glucoside, lonicerin, tricin, etc. (Choi et al., 2007; Lee et al., 2010; Ge et al., 2019; Fang et al., 2020). Other flavonoids including one flavonolignan (hydnocarpin), one flavanone (eriodictyol) and three biflavonoids [3'-O-methyl loniflavone (5,5'',7,7''-tetrahydroxy 3'-methoxy4',4'''-biflavonyl ether), loniflavone (5,5'',7,7'',30-pentahydroxy 4',4'''-biflavonyl ether) and (5,7,8,4'-tetrahydroxyflavone)-3'-4-(5,7-dihydroxyflavone)] were also have been isolated and identified from *L. japonica* (Kumar et al., 2005; Ge et al., 2018; Li et al., 2019). According to modern pharmacological research, flavonoids extracted from *L. japonica* has health benefits for the prevention of cancer, diabetes, cardiovascular disease, liver injury, and cerebrovascular disease (Han et al., 2016; Ge et al., 2018; Wan H. et al., 2019).

### Saponins

Most of saponins from *L. japonica* belong to the oleanane type and hederagenin type (Shang et al., 2011). Saponins in *L. japonica* were first studied by Kawai et al. (1988), and 15 chemical compounds were found. So far, about 30 saponins, such as α-Hederin, Loniceroside A–E have been isolated and identified from *L. japonica* (Kawai et al., 1988; Son et al., 1994; Choi et al., 2007; Lin et al., 2008; Qi et al., 2009; Shang et al., 2011; Kuroda et al., 2014; Yu et al., 2015; Wang L. et al., 2016; Li et al., 2019). Studies showed that saponins from *L. japonica* have anti-inflammatory activities *in vitro* and *in vivo* (Lee et al., 1995; Kwak et al., 2003; Li et al., 2017; Ge et al., 2019).

### Iridoids

Iridoids are the most abundant compounds in *L. japonica*, which mostly presenting as glycosides (Wang L. et al., 2016; Li et al., 2019). So far, more than 92 iridoids, like loganin, sweroside, secologanoside, ethyl secologanoside, centauroside etc., have been isolated from *L. japonica* (Kakuda et al., 2000; Yu et al., 2011, 2013; Zheng et al., 2012; Kashiwada et al., 2013; Liu et al., 2015, 2020; Ge et al., 2019; Yang R. et al., 2020; Qiu et al., 2021). Studies showed that these iridoids have anti-inflammatory (Song et al., 2008; Yu et al., 2011; Qiu et al., 2021) and antiviral activities (Kashiwada et al., 2013; Yu et al., 2013) *in vitro* and *in vivo*.

### Others

Other chemical components except for phenolic acids, essential oils, flavonoids, saponins, and iridoids have also been isolated from *L. japonica*. Zhao et al. (2018) had identified 13 trace elements (Mg, Cr, Mn, Fe, Ni, Cu, Zn, As, Se, Mo, Cd, Hg, and Pb) with inductively coupled plasma mass-spectrometry (ICP-MS) and high-performance liquid chromatography-photodiode array (HPLC-PDA) method. Cai et al. (2019, 2021) had identified 13 amino acids (Alanine, Serine, Proline, Valine, Threonine, Isoleucine, Leucine, Aspartic acid, Glutamate, Lysine, Histidine, Phenylalanine, and Arginine) and four nucleosides (Cytidine, Uridine, Adenosine, and Inosine) in *L. japonica*.

## *Lonicera Japonica* (Extracts) and Intestinal Antioxidant

In addition to its medicinal uses, *L. japonica* is also widely used in healthy foods and cosmetics in the world because of its health benefits (Seo et al., 2012; Fang et al., 2020; Zhang T. et al., 2020). Modern pharmacological researches have demonstrated that *L. japonica* extract has a variety of biological activities, which the antioxidant activity is an important biological property of great interest (Hsu et al., 2016; Wan H. et al., 2019; Zhang T. et al., 2020). Antioxidant activity of *L. japonica* was mainly related to its abundant polyphenols (Lee et al., 2019) and polysaccharides (Zhou et al., 2020).

### Antioxidant Activity of Polyphenols

**Figure 1:**
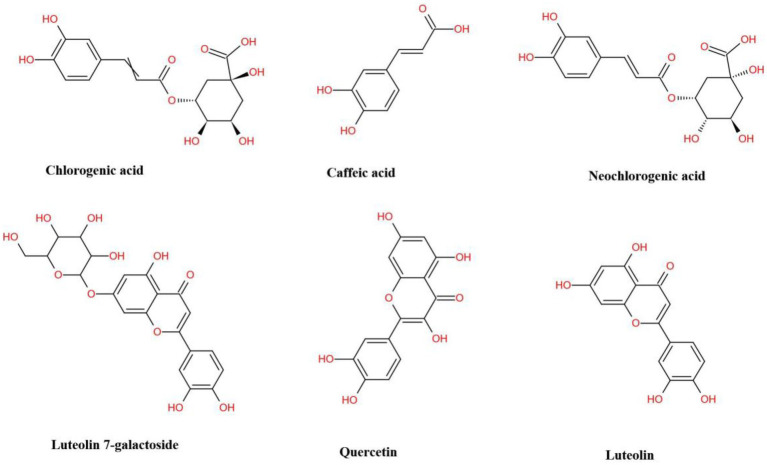
The structural formula of major phenolic acids and flavonoids presenting in *Lonicera japonica*.

The antioxidative property of *L. japonica* is mainly attributed to the specific chemical structure of polyphenols, a widespread group of secondary metabolites that include various phenolic acids and flavonoids, which have a common character of having at least one aromatic ring substituted with one or more hydroxyl groups (Kong et al., 2017; Fan et al., 2019). Lee et al. (2019) who reported that the antioxidant activities of *L. japonica* were positively correlated with total phenolic, total flavonoid, CGA, CA, and quercetin contents, and Kong et al. (2017) who reported that antioxidative activity of *L. japonica* presented a significant positive correlation with the content of CGA, cynaroside, rutin, and hyperoside can demonstrate this conclusion. Figure 1 presented the main phenolic acids (GCA, CA, and NGA) and flavonoids (luteolin 7-galactoside, quercetin, and luteolin) in *L. japonica*. It showed that all these compounds contain an aromatic nucleus and hydroxyl group, which is related to their strong antioxidant capacity (Choi et al., 2007; Guo et al., 2014; Hsu et al., 2016).

The ability to scavenge free radicals may play an important role in preventing some diseases caused by free radicals (Gheisar and Kim, 2018). Normally, 1,1-diphenyl-2-picrylhydrazyl (DPPH) scavenging activity assay, 2,2'-azino-bis (3-ethylbenzothiazoline-6-sulfonic) acid (ABTS) scavenging activity assay, superoxide radical scavenging activity assay, ferric-reducing antioxidant power (FRAP) assay, and reducing power (RP) assay are the most frequently used to evaluate the antioxidant activity of plant extracts (Lee et al., 2011, 2019; Kong et al., 2017; Zhang T. et al., 2020). DPPH radical scavenging activity and ABTS radical scavenging activity reflect the ability of hydrogen-donating antioxidants and electron transfer to scavenge DPPH and ABTS^+^ radicals (Lee et al., 2019). Superoxide radical scavenging activity denotes the ability to remove free radicals, such as peroxyl, alkoxyl, hydroxyl, and nitric oxide, which formed from superoxide anions through the Fenton reaction, lipid oxidation, or nitric oxidation (Hsu et al., 2016). FRAP and RP assays represent their ability to reduce the of ferric (Fe^3+^) form to the ferrous (Fe^2+^) form (Seo et al., 2012; Lee et al., 2019). Chaowuttikul et al. (2017) reported that the ethanolic extract of *L. japonica* showed DPPH and nitric oxide scavenging activities as well as RP property. Lee et al. (2019) showed that DPPH and ABTS radical scavenging activity of *L. japonica* were significantly increased during 60 min of heating and were retained for 90 min.

### Antioxidant Activity of Polysaccharides

Polysaccharides are a kind of natural polymer linked by aldose or ketose through glycosidic bonds (Zhou et al., 2018a, 2021). Previous studies have found that polysaccharides extracted from plants can relieve oxidative stress through exerting their antioxidation potentials (Surin et al., 2018; Zhou et al., 2020). Polysaccharide is one of the main active ingredients of *L. japonica*, which have been isolated and identified in previous studies (Zhou et al., 2018a, 2020; Liu et al., 2019; Zhang T. et al., 2020). *In vitro* study showed that polysaccharide extracts from *L. japonica* exhibited obvious DPPH-scavenging activity, ABTS^+^-scavenging activity, hydroxyl radical-scavenging activity, superoxide radical-scavenging activity, and excellent inhibitory activity on erythrocyte hemolysis induced by H_2_O_2_ (Zhang T. et al., 2020). Polysaccharide extracts from *L. japonica* could protect cardiomyocytes of mice injured by hydrogen peroxide *via* increasing the activities of catalase (CAT), glutathione peroxidase (GSH-Px), and superoxide dismutase (SOD), and decreasing ROS production (Zhou et al., 2020). *In vivo* study showed that crude polysaccharides extracted from *L. japonica* could alleviate the oxidative damage of liver in streptozotocin (STZ)-induced diabetic rats by decreasing alanine aminotransferase (ALT), aspartate aminotransferase (AST), and gamma-glutamyl transpeptidase (GGT) in serum, and improving levels of CAT, SOD, and GSH in liver (Wang et al., 2017). It reveals that the polysaccharides play an important role in the antioxidant function of *L. japonica*.

### Potential Intestinal Antioxidant Effects of *L. japonica* (Extract)

Reactive oxygen species (ROS) are generated along with the process of cell respiration and normal metabolism continuously, and mitochondrion is the primary source of the majority of ROS in organisms (Wang Y. et al., 2020; Yan Z. et al., 2020). ROS includes free radical ROS and non-radical ROS. Free radical ROS mainly include superoxide anion free radicals (O_2_^−^), hydroxyl radical (·OH^−^), peroxyl radical (ROO), and alkoxyl radical (RO), and non-radical ROS mainly consist of hydrogen peroxide (H_2_O_2_), oxygen (O_2_), ozone (O_3_), hypochlorous acid (HOCL), hypobromous acid (HOBr), chloramines (RNHCL), and organic hydroperoxides (ROOH; Wang Y. et al., 2020). Under normal physiological conditions, ROS can act as signaling molecules involved in cell growth and cellular adaptive responses (Lum and Roebuck, 2001). However, in commercial animal production, animals often suffer from bacterial infection (Zhang X. et al., 2020), endotoxin challenge (Chen et al., 2021), mycotoxin challenge (Xu et al., 2020), and weaning stress (Zhou et al., 2018b), which may induce a large number of ROS. When the body cannot remove these ROS in time, oxidative stress injury occurs (Campbell et al., 2013; Yin et al., 2014; Zhu H. et al., 2018; Saracila et al., 2021). Numerous studies have demonstrated that oxidative stress is associated with many pathological conditions, including intestinal barrier dysfunction and various digestive tract diseases (Almenier et al., 2012; Navarro-Yepes et al., 2014; Cao et al., 2018; Tang et al., 2018b; Chen et al., 2020, 2021). Thus, alleviating the negative effects of oxidative stress damage is crucial for the development of the animal husbandry.

The latest research progress of antioxidant activity of *L. japonica* has been summarized in Table 1. These studies suggested that *L. japonica* might be potential natural antioxidants and beneficial chemopreventive agent, which can be inferred that the extract of *L. japonica* may have a protective effect on intestinal oxidative damage of animals. However, the direct evidence of the protective effects *L. japonica* on intestinal oxidative damage is still lack. Therefore, further studies are needed to confirm whether *L. japonica* have a regulating effect on the intestinal oxidative damage of animals including farm animals and aquatic animals.

**Table 1:** Antioxidant activity of *Lonicera japonica in vitro* and *in vivo*.

Animal/Cell models	Active compounds	Main results	References
LPS-induced RAW264.7 cells	Ethanolic extract	Significantly decreased the ROS level in the stimulated macrophage cells	Yoo et al., 2008
6-OHDA-induced SH-SY5Y cells	Ethyl acetate extract	Significantly decrease ROS and increase the GSH level, SOD activity, and CAT activity in 6-OHDA-induced SH-SY5Y cells	Kwon et al., 2012
H_2_O_2_-induced rat cardiomyocytes	Caffeoylquinic acids	Significantly attenuated hypoxia-induced ROS generation and reduced the ratio of GSSG/GS total	Wang C. et al., 2016
High-fat-induced hyperlipidemia rats	Water extracts	Could suppress the oxidative stress by increasing serum SOD, GSH-Px, and reducing MDA concentration in hyperlipidemia rats	Wang F. et al., 2016
Streptozotocin (STZ)-inducd diabetic rats	Polysaccharide	The oxidant stress in liver was restored by increasing the levels of CAT, SOD, and GSH in liver	Wang et al., 2017
H_2_O_2_-induced HepG 2 cells	Flavonoids	Dose-dependent increased CAT and SOD activity	Tzeng et al., 2014
H_2_O_2_-induced RAW264.7 cells	Flavonoids	Dose-dependent reduced MDA content in cells and culture supernatant, improve SOD activity and GSH content, and increase intracellular lactate dehydrogenase activity.	Luo et al., 2018
Carbon tetrachloride-induced liver fibrosis mice	Water extract	Alleviated liver oxidative stress injury and enhanced the activation of Nrf2 anti-oxidant signaling pathway	Miao et al., 2019
H_2_O_2_-induced hepatoma cells	Japoflavone D	Treatment of Japoflavone D suppressed the activation of ERK and mTOR and activated the KEAP1/NRF2/ARE signaling axis	Wan H. et al., 2019
Gastritis and peptic ulcer rats	BST-104	BST-104 treatment increased antioxidant activities (higher levels of CAT, SOD, and GSH/GSSG, and lower MDA levels)	Bang et al., 2019
H_2_O_2_-induced HepG2 cells	4,5-CQME	Reduced ROS and MDA levels and rescued GSH depletion; 4,5-CQME regulated the Keap1/Nrf2 signaling pathway and enhanced both the mRNA and protein expressions of HO-1 and NQO1	Xiao et al., 2020
H_2_O_2_-induced mice cardiomyocytes	Polysaccharide	Significantly increased the activities CAT, GSH-Px, and SOD, and decrease ROS production	Zhou et al., 2020
Beef cattle under heat stress	Not mentioned	Serum SOD, GSH-Px, and T-AOC was increased, and serum MDA was decreased	Fu et al., 2016
Dairy cows	Not mentioned	Quadratically increased the activity of GSH-Px and T-AOC in serum but decreased concentration of MDA	Ma et al., 2020a
Dairy cows	Not mentioned	*Lonicera japonica* supplementation decreased the concentrations of reactive ROM, meanwhile increased the T-AOC and SOD concentrations in blood	Zhao et al., 2020

BST-104, a water extract of *L. japonica*; LPS, lipopolysaccharide; CAT, catalase; GSH-Px, glutathion peroxidase; SOD, superoxide dismutase; T-AOC, total antioxidant capacity; MDA, malonaldehyde; ROM, reactive oxygen metabolites; 6-OHDA, 6-hydroxydopamine; rCMEC, rat cardiac microvascular endothelial cells; ROS, reactive oxygen species; 4,5-CQME, 4,5-di-O-caffeoylquinic acid methyl ester; Keap1, kelch-like ECH-associated protein 1; Nrf2, nuclear factor erythroid 2-related factor 2; ARE, antioxidant response elements; HO-1, heme oxygenase; NQO1, quinone oxidoreductase; ERK, extracellular signal-related kinases; and mTOR, mammalian target of rapamycin protein.

## *Lonicera Japonica* (Extracts) and Intestinal Inflammation

The intestine is different from the other organs of animal because it is not only the main part of animal nutrition digestion and absorption, but also consists of a physical and immunological protective barrier against foreign antigens and pathogens from the external environment into the circulation system (Tang et al., 2016, 2018b, 2021; Curciarello et al., 2019). Optimum intestinal health is of prime importance to animal growth as well as animal health. Disruption of the intestinal epithelial homeostasis has been reported to increase intestinal permeability, which can cause numerous gastrointestinal diseases (Miner-Williams and Moughan, 2016; Tang et al., 2019; Peng et al., 2020; Tang and Xiong, 2021). *Lonicera japonica* extract has a significant effect on the intestinal health regulation of animals due to its various biological activities including anti-inflammatory activity (Kang et al., 2010; Han et al., 2016; Zhou et al., 2021).

### Anti-inflammatory Activity of *L. japonica* (Extracts)

Inflammation is a normal protective response induced by tissue injury or infection. It has been proved that *L. japonica* presents significant anti-inflammatory effects *in vitro* and *in vivo* (Jiang et al., 2014; Li R. et al., 2020; Zhou et al., 2021). As we know, proinflammatory cytokines, such as tumor necrosis factor α (TNF-α), interleukin 1β (IL-1β), and IL-6 contribute to inflammatory injury and triggers an inflammatory cascade (Bang et al., 2019; Li R. et al., 2020). Kang et al. (2010) showed that *L. japonica* extract could suppress inflammatory mediators, such as IL6, IL-8, and TNF-α release by blocking nuclear factor-κB (NF-κB) and mitogen-activated protein kinase (MAPKs) activation pathways in HMC-1 Cells. Su et al. (2021) showed that ethanol extract of *L. japonica* caulis significantly inhibit the expression of pro-inflammatory factors such as TNF-α, IL-1β, IL-6, and interferon γ (IFN-γ) in mice. The study Li R. et al. (2020) suggested that the flower buds, leaves, and stems of *L. japonica* extracts showed a cytoprotective effect on lipopolysaccharide (LPS) stimulated RAW 264.7 macrophages by suppressing proinflammatory cytokines including TNF-α, IL-1β, and IL-6 production. Bang et al. (2019) showed that the anti-inflammatory effects of BST-104 (a water extract of *L. japonica*) were attributed to reduced levels of proinflammatory cytokines, such as TNF-α, IL-1β, and IL-6 in gastric mucosal tissues. All of these researches suggest that *L. japonica* is a good anti-inflammatory agent for treating inflammatory disorders.

### *Lonicera japonica* (Extracts) Inhibits Intestinal Inflammation

The intestinal tract is the largest immune organ in the body and acts as the first line of defense against infection and a barrier that prevents commensal bacteria from penetrating the intestinal epithelium (Tang et al., 2016; Clavijo and Flórez, 2018; Qamar et al., 2021). The gut immune system comprises mucosal layer, epithelial cells, antibacterial peptides, immunoglobulins, and cytokines (Yitbarek et al., 2019; Qamar et al., 2021). Previous studies had demonstrated that *L. japonica* can promote intestinal immune function and has a preventive effect on intestinal inflammation (Park et al., 2012; Yang X. et al., 2020). Yang X. et al. (2020) showed that the treatment of the alcohol extract of *L. japonica* to mice significantly increased intestinal sIgA content. Zhou et al. (2021) showed that with the supplementation of *L. japonica* polysaccharides, the content of immunoglobulin A (sIgA) secreted from the intestine was significantly higher than that of dextran sulfate sodium (DSS)-induced ulcerative colitis mice. sIgA, an immunoglobulin secreted by plasma cells of the intestinal mucosa, is a major effector of the intestinal mucosal immunity, which acts as the first line of defense in the intestinal mucosa that neutralizes pathogens in the intestinal mucosa and plays an important role in local anti-infection of the body (Salerno-Goncalves et al., 2016; Zhou et al., 2021). These studies indicated that prompting the secretion of sIgA is one of the ways to enhance the immune ability of the intestine by *L. japonica* (Yang X. et al., 2020; Zhou et al., 2021). In addition to promoting the secretion of sIgA, *L. japonica* can also play the role of intestinal immune function by regulating the secretion of intestinal mucosal cytokines (Park et al., 2012; Zhou et al., 2018a). Lee et al. (2011) showed that buthanol (BuOH) extracts of *L. japonica* inhibited the synthesis of IL-6 in a LPS-stimulated colonic epithelial cell line (HT-29 cell) *in vitro* and a DSS-induced ulcerative colitis mouse *in vivo*. Park et al. (2012) showed that *L. japonica* inhibited the cytokines including TNF-α, IL-1β, IL-6, IFN-γ, IL-12, and IL-17 in DSS-induced ulcerative colitis mice. In an immunosuppressed mice model, the researchers found that polysaccharide extracts from *L. japonica* could restore the levels of serum cytokines IL-2, TNF-α, and IFN-γ level in cyclophosphamide-induced mice, which indicated that *L. japonica* can be used as a potential immunomodulatory agent (Zhou et al., 2018a). Through these studies, we can speculate that *L. japonica* extract may also had regulation on intestinal immune function and intestinal inflammation of farm and aquatic animals, which of course needs further researches to demonstrate it.

## *Lonicera Japonica* (Extracts) and Gut Microbiota

### Gut Microbiota and Intestinal Health

**Figure 2:**
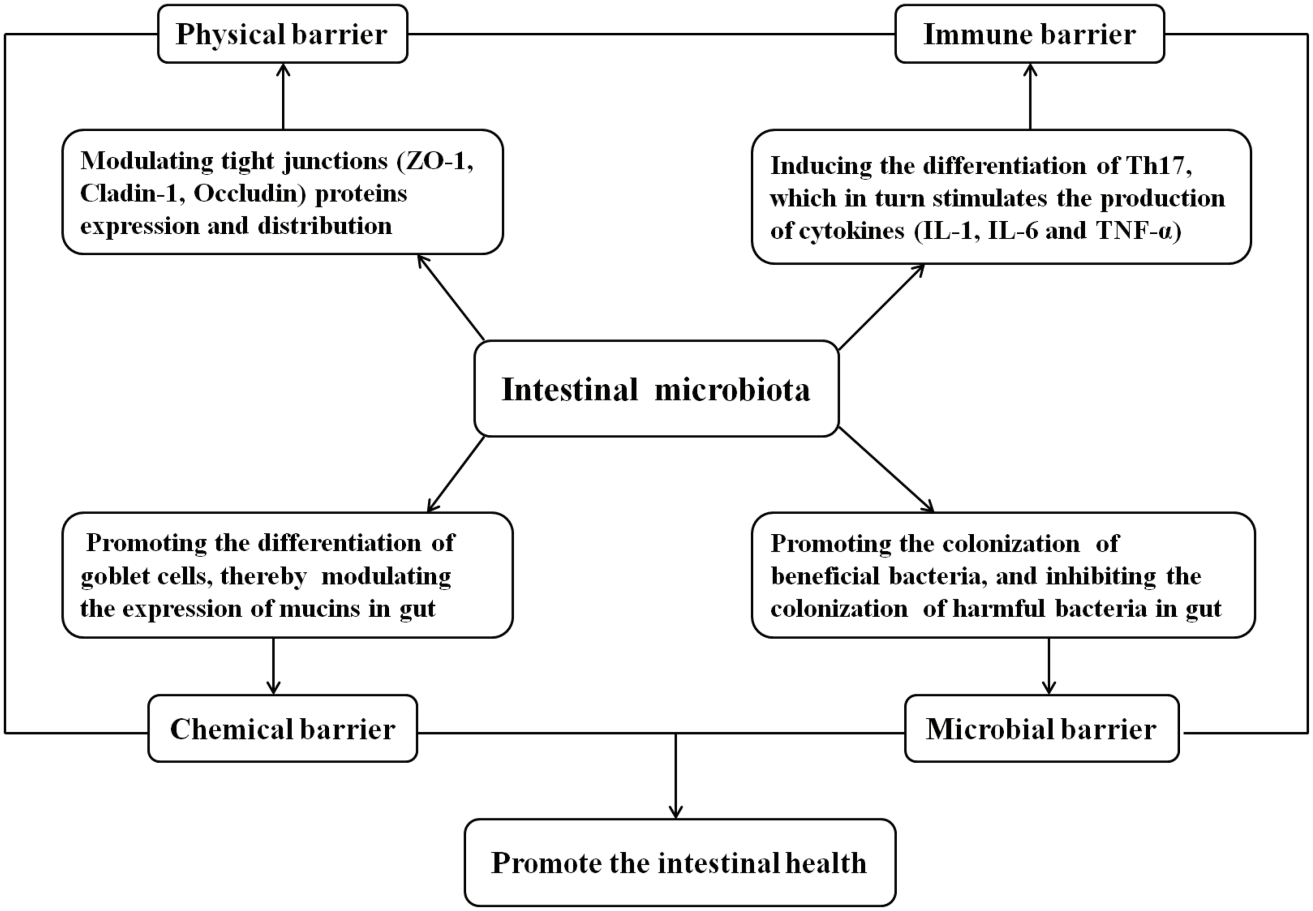
Relationship between gut microbiota and intestinal health.

The gastrointestinal tract, the largest organ in the animal body, provides a broad colonization surface for the flora. Thousands of bacteria colonize the entire gut, which directly interrelates with the host and contributes to the regulation of the host intestinal barrier function and homeostasis (Chen et al., 2017b; Wan M. et al., 2019; Hayashi et al., 2021; Qamar et al., 2021). The gut barrier is central to the maintenance of gut homeostasis and breakdown of the barrier is involved in a wide variety of clinical conditions (Alam and Neish, 2018). Gut microbiota plays a vital role in host health, which is thought to tightly associate with the intestinal barrier function including physical barrier, chemical barrier, immune barrier, and microbial barrier (Figure 2; Guevarra et al., 2018; Makki et al., 2018). First of all, the intestinal microbial barrier is composed of many normal intestinal floras, which play an important role in intestinal microecological balance regulation, and the imbalance of intestinal floras may result in intestinal dysfunction (Tan et al., 2015; Li X. et al., 2020). Second, intestinal commensal segmented filamentous bacteria can induce the differentiation of T helper 17 (Th17) in the lamina prima, which in turn stimulates the production of cytokines, IL-1, IL-6, and TNF-α by a variety of cells (Goto et al., 2014; Villena et al., 2014). Metabolites such as short-chain fatty acids (SCFA) produced by the gut bacteria are considered as key molecular intermediates between the microbiota and its host (Beaumont et al., 2020). SCFA can induce the proliferation and differentiation of Treg, thus activating the intestinal immune system and playing the function of immune barrier (Horai et al., 2017). Third, the intestinal floras can influence the intestinal physical barrier by modulating tight junction (TJ) proteins expression and distribution (Zhu L. et al., 2018; Li X. et al., 2020). For example, Hu et al. (2020) reported that piglets receiving protocatechuic acid promoted the expression of ZO-1 and Claudin-1 in the intestinal mucosa by increasing the abundance of the beneficial bacteria *Roseburia* in the intestinal tract thus maintaining the function of the intestinal barrier. Finally, the intestinal floras can also influence the intestinal chemical barrier by promoting the differentiation of goblet cells, thereby modulating the expression of mucins (MUCs), a family of highly glycosylated protein that are secreted by specialize cells in the gut, which is the main component of intestinal mucus (Sicard et al., 2017). In a word, intestinal flora is closely related to intestinal health.

### *Lonicera japonica* (Extracts) Modulates Intestinal Micobiota

Modern pharmacological research has confirmed the strong antimicrobial activity of *L. japonica in vivo* and *in vitro* (Rhee and Lee, 2011; Xiong et al., 2013; Yang et al., 2016; Minami and Makino, 2020; Yan L. et al., 2020). *In vitro* study showed that *L. japonica* has antimicrobial effects such as *Bacteroides fragilis*, *Bacteroides ovatus*, *Clostridium difficile*, *Clostridium perfringenes*, *Propionebacterium acnes*, *Staphylococcus aureus*, *Shigella*, *Salmonella*, and *Escherichia coli* (*E. coli*; Rhee and Lee, 2011; Xiong et al., 2013; Yang et al., 2016, 2018; Yan L. et al., 2020). *In vivo* study showed that *L. japonica* could significantly promote the colonization of beneficial bacteria and inhibit the reproduction of harmful bacteria (Minami and Makino, 2020; Yang X. et al., 2020). Wang et al. (2014) showed that unfermented or fermented *L. japonica* both can significant alteration of the distribution of intestinal flora, especially affecting the population of *Akkermansia* spp. and Bacteroidetes/Firmicutes ratio in obesity rats, which play an essential role in high fat diet or LPS induced enhancement in gut permeability, development of endotoximia, and inflammation. Minami and Makino (2020) showed that *L. japonica* significantly increased the survival rate and decreased *Citrobacter rodentium* (*C. rodentium*) colonization in the large intestine of mice. *Citrobacter rodentium* is a mucosal pathogen of murine, which has long used as a model to elucidate the molecular and cellular pathogenesis of infection with enteropathogenic *E. coli* and enterohaemorrhagic *E. coli*, two clinically important human gastrointestinal pathogens (Collins et al., 2014; Bouladoux et al., 2017; Mullineaux-Sanders et al., 2019). Yang X. et al. (2020) showed that the water extract of *L. japonica* and alcohol extract of *L. japonica* did not damage the intestinal structure, and both of them could promote the growth of beneficial bacteria *Lactobacillus* and inhibit the growth of potential pathogenic bacteria *E. coli*. *Lactobacillus* is a predominant indigenous bacterial genus found in the human and animal gastrointestinal tract, and species of this genus like *Lactobacillus plantarum* (*L. plantarum*; Wang et al., 2018), *Lactobacillus casei* (Eun et al., 2011), *Lactobacillus rhamnosus* (Villena et al., 2014), and *Lactobacillus reuteri* (Yang et al., 2015) etc., are commonly used as probiotics, which can affect transepithelial electrical resistance (TER) and epithelial permeability, modulate TJ proteins distribution, and enhance the immune function. *Escherichia coli* strains are important pathogens that cause diverse diseases in humans and animals, which is a major challenge for intestinal health (Dautzenberg et al., 2016; Stromberg et al., 2017; Desvaux et al., 2020). Therefore, it reveals that *L. japonica* has a good regulatory effect on animal intestinal microbiota, thus promoting the intestinal health of animals. However, the studies of *L. japonica* extract on intestinal microbiota of farm and aquatic animals are still lack, which perhaps is a good research direction in the future.

## Application of *L. Japonica* in Animal Production

Various herbs and their extracts have been used as feed additives due to their anti-oxidative effect, anti-inflammatory activity, anti-microbial effect, and growth-promoting effect (Windisch et al., 2008; Hanczakowska et al., 2015; Lei et al., 2018; Lin et al., 2020). Among them, *L. japonica* (extract) was widely investigated in animal husbandry because of its diverse pharmacological effects such as antioxidant, anti-microbial, antiviral, antitoxic, antiseptic, and anti-inflammatory properties (Kang et al., 2010; Park et al., 2012; Wang et al., 2014; Li R. et al., 2020). Table 2 summarized the application of *L. japonica* (extract) in animal production in recently years. It showed that these studies mainly focus on pigs (Liu W. et al., 2016), beef cattle (Fu et al., 2016; Yejun et al., 2019), dairy cows (Ma et al., 2020a,b; Zhao et al., 2020), broiler (Müştak et al., 2015), laying hens (Long et al., 2018), *Penaeus monodon* (Chen et al., 2013), grass carp (Meng et al., 2019), and olive flounder (Dharaneedharan et al., 2016).

**Table 2:** Application of *L. japonica* in animal production.

Animals	*In vivo*/*In vitro*	Optimal added amount	Significant effects	References
Beef cattle	*In vivo*	0.2% in concentrate	Dietary supplementation of *L. japonica* extract improved the antioxidant and restored the morphosis of damaged muscle	Song et al., 2015
Beef cattle	*In vivo*	0.2% in concentrate	Dietary supplementation of *L. japonica* extracts improve antioxidant capability, and relieve stress reaction of beef cattle, while had no significant effects on weight gain	Fu et al., 2016
Beef cattle	*In vitro*	3, 5, 7, and 9%	*Lonicera japonica* extract supplementation could significantly reduce rumen methane (CH4) production as well as inhibit fiber-decomposition bacteria and methanogens	Huang et al., 2019
Beef cattle	*In vitro*	3, 5, 7, and 9%	*Lonicera japonica* extract supplementation linear decreased gas production and dry matter degradability, decreased CH4 production, and fibrolytic bacteria and ciliate associated methanogen abundance	Yejun et al., 2019
Dairy cow	*In vivo*	28 g/d	Dietary supplemented with *L. japonica* extract could relieve heat stress of dairy cows without affect the performance of lactating cows as well as cause changes of hepatic gene expression, such as immune, antioxidant capacity, and liver glucose metabolism related genes	Gao et al., 2021
Dairy cow	*In vivo*	28 g/d	Dietary *L. japonica* extract supplementation had no significant effect on the performance of cows under heat stress, but can improve the immune response and alleviate the heat stress of cows	Ma et al., 2020b
Dairy cow	*In vivo*	28 g/d	Dietary *L. japonica* extract supplementation had no negative effects on lactation performance but helped to alleviate heat stress by improving antioxidant status and promoting endocrine and immune functions	Ma et al., 2020a
Dairy cow	*In vivo*	1 and 2 g/kg dry matter	Supplementation with 1 and 2 g/kg dry matter *L. japonica* extract could improve lactation performance, increase milk production, and enhance anti-inflammatory and antioxidant capacities of dairy cows during perinatal period	Zhao et al., 2020
Dairy cow	*In vitro*	1 mg/g	Supplementation with *L. japonica* extract can effectively regulate the fermentative state of rumen microorganism under the *in vitro* condition	Tang et al., 2018a
Pig	*In vivo*	0.025 and 0.05% herbal extract mixture (HEM)^1^	Administration of HEM (0.025 and 0.05%) could improve growth performance and nutrient digestibility, decrease serum cortisol levels, as well as benefit the meat quality in finishing pigs	Liu W. et al., 2016
Pig	*In vivo*	1,000 mg/kg HEM^2^	Supplementation with *L. japonica* extract had benefit effects on intestinal morphology modulation and the mRNA expression of nutrients transporters	Wang Y. et al., 2020
Broiler	*In vivo*	0.3 and 1% HEM^3^	Supplementation with *L. japonica* extract did not affect proximate composition of the breast meat, but could increase total phenols content of the breast meats; could increase the antioxidative potential and overall preference of breast meat during cold storage	Jang et al., 2008
Broiler	*In vivo*	0.2%	Supplementation with *L. japonica* extract increased weight gain, blood cells, antioxidant activity, and meat quality of broilers	Park et al., 2014
Broiler	*In vivo*	190 μg/d	Supplementation with *L. japonica* extract could improve the live body weight as well as decrease *Mycoplasma gallisepticum* colonization of broilers	Müştak et al., 2015
Laying hens	*In vivo*	0.025 and 0.05% HEM^4^	Supplementation with HEM could improve eggshell strength and shelf life in laying hens when reared under hot climatic conditions.	Liu and Kim, 2017
Laying hens	*In vivo*	300 mg/kg	Supplementation with *L. japonica* extract could increase the average egg weight, average daily feed intake, and egg Haugh unit, improve the lipid metabolism, and reduce cholesterol content of egg yolk	Long et al., 2018
*Penaeus monodon*	*In vivo*	0.2 and 0.4%	Supplementation with *L. japonica* extra could improve the growth performance, health condition, and survival rate of *Penaeus monodon*	Chen et al., 2013
Grass carp	*In vivo*	20 and 40 g/kg	Supplementation with *L. japonica* extract could effectively improve the lipid metabolism and ameliorate the lipid deposition of grass carp	Meng et al., 2019
Olive flounder	*In vivo*	0.025, 0.05, 0.1, 0.2, and 0.4%	Fish fed with *L. japonica* leaf powder showed decreased cumulative mortality and enhanced immunity response and resistance to *Vibrio anguillarum* infection	Dharaneedharan et al., 2016

1 A mixture of 55% *Scutellaria baicalensis* powder extract, 25% *L. japonica* powder extract, and 20% carrier (wheat bran).

2 A mixture extract of golden-and-silver honeysuckle (*L. japonica* Thunb.), huangqi (*Astragalus menbranaceus*), duzhong leaves (*Eucommia folium*), and dangshen (*Codonopsis pilosula*).

3 A mixture of mulberry leaf, Japanese honeysuckle, and goldthread at a ratio of 48.5:48.5:3.0.

4 A mixture of 55% *S. baicalensis* powder extract, 25% *L. japonica* powder extract, and 20% carrier (wheat bran).

Studies on beef cattle showed that *L. japonica* extract can effectively alleviate heat stress, improve antioxidant function, and have a good repair effect on skeletal muscle fiber structure damage of beef cattle (Song et al., 2015; Fu et al., 2016). Moreover, *in vitro* studies showed that *L. japonica* extract could regulate rumen fermentation and reduce methane production by inhibiting the growth of methanogenic bacteria (Huang et al., 2019; Yejun et al., 2019). Studies on dairy cows showed that dietary supplementation of *L. japonica* extract could relieve heat stress of dairy cows by improving immune and antioxidant capacity (Ma et al., 2020a,b; Gao et al., 2021), enhancing anti-inflammatory activity (Zhao et al., 2020). Meanwhile, *L. japonica* extract can improve rumen microbial diversity and improve rumen fermentation capacity (Tang et al., 2018a). In pig production, herbal extract mixture (HEM) may have a better application effect (Liu W. et al., 2016; Wang M. et al., 2020). For instance, Liu W. et al. (2016) indicated that dietary supplementation with a mixture of 55% *Scutellaria baicalensis* extract and 25% *L. japonica* extract administration could improve growth performance and nutrient digestibility, decrease serum cortisol levels, as well as benefit the meat quality in finishing pigs, and Wang Y. et al. (2020) showed that dietary supplementation with 1,000 mg/kg a mixture extract of golden-and-silver honeysuckle (*L. japonica* Thunb.), huangqi (*Astragalus menbranaceus*), duzhong leaves (*Eucommia folium*), and dangshen (*Codonopsis pilosula*) had beneficial effects on intestinal morphology modulation and the mRNA expression of nutrients transporters of pigs. For broilers, dietary supplementation with *L. japonica* extract could increase weight gain, blood cells, antioxidant activity, and meat quality of broilers (Park et al., 2014), while did not affect the proximate composition of the breast meat, but could increase the antioxidative potential and overall preference of breast meat during cold storage (Jang et al., 2008). Drinking water containing GCA extracted from *L. japonica* can effectively increase the body weight of broilers and reduce Mycoplasma gallisepticum infection of broilers (Müştak et al., 2015). Studies on laying hens showed that dietary supplementation with *L. japonica* extract (Long et al., 2018) or HEM containing *L. japonica* extract (Liu and Kim, 2017) could improve laying performance, eggshell strength, egg quality, and shelf life in laying hens. For aquatic animals, dietary supplementation with *L. japonica* could improve the growth performance, health condition and survival rate of *Penaeus monodon* (Chen et al., 2013), and could effectively improve the lipid metabolism and ameliorate the lipid deposition of grass carp (Meng et al., 2019). Flounder fish fed with 0.025, 0.05, 0.1, 0.2, and 0.4% *L. japonica* leaf powder for 4 weeks showed significantly increased respiratory burst, lysozyme, phagocytic activity, immune function, and antioxidant activity (Dharaneedharan et al., 2016).

## Conclusion

Intestinal health determines the health status of animals. To regulate intestinal health is always been an important issue in the post-antibiotic era of animal husbandry. As a kind of natural plant extract, *L. japonica* extract is rich in phenolic acids, essential oils, flavonoids, iridoids, and saponins, which has a good regulating effect on the intestinal health of animals, and is an ideal product of antibiotics substitution. According to the published literature, although the application of *L. japonica* extract in animal production has been reported, it mainly focuses on the regulation of animal production performance, meat quality, egg quality, rumen fermentation capacity, and anti-heat stress, etc. In animal production, the effects of *L. japonica* extract on intestinal health may be related to its antioxidant, anti-inflammatory, and antimicrobial activities. Although previous studies had demonstrated that about *L. japonica* has a good regulatory effect on animal intestinal health, but mainly focus on experimental animals or cells, like mice, rats, HMC-1 Cells, and RAW 264.7 cells, the studies of *L. japonica* extract on intestinal health regulation of farm and aquatic animals are still rare and unclear. Therefore, it is necessary to increase the research on the regulatory mechanism of *L. japonica* extract on intestinal health especially the protective effects of *L. japonica* extract on oxidative injury, inflammation, and regulation of intestinal flora in farm and aquatic animals in the future, so as to provide a theoretical basis for the application of *L. japonica* extract in animal production.

## Author Contributions

XT and RF advocated to writing this review, and reviewed, edited, and approved its final version. XT collected literature and wrote the manuscript. XL and JZ helped to collect and review literatures. All authors contributed to the article and approved the submitted version.

## Conflict of Interest

The authors declare that the research was conducted in the absence of any commercial or financial relationships that could be construed as a potential conflict of interest.

## Publisher’s Note

All claims expressed in this article are solely those of the authors and do not necessarily represent those of their affiliated organizations, or those of the publisher, the editors and the reviewers. Any product that may be evaluated in this article, or claim that may be made by its manufacturer, is not guaranteed or endorsed by the publisher.
